# Effects of Whole-Body 50-Hz Magnetic Field Exposure on Mouse Leydig Cells

**DOI:** 10.1100/tsw.2004.182

**Published:** 2004-10-20

**Authors:** Zsolt Forgács, Zoltán Somosy, Györgyi Kubinyi, Hanna Sinay, Jozsef Bakos, György Thuróczy, András Surján, Aranka Hudák, Ferenc Olajos, Péter Lázár

**Affiliations:** ^1^ National Institute of Chemical Safety, Budapest, Hungary; ^2^ “National Frederic Joliot-Curie” Research Institute for Radiobiology and Radiohygiene, Budapest, Hungary; ^3^ Clinical Laboratory, National Institute of Occupational Health, Budapest, Hungary; ^4^ Second Department of Gynecology, Semmelweis University, Budapest, Hungary

**Keywords:** ELF magnetic fields, power-line frequency, testosterone, Leydig cell culture, radioimmunoassay, hCG, histology, hematology, serum chemistry

## Abstract

The main goal of this study was to evaluate the possible effect of whole-body magnetic field (MF) exposure on the steroidogenic capacity of Leydig cells *in vitro*. In four separate experiments, male CFLP mice were exposed to sinusoidal 50-Hz, 100-μT MF. The duration of exposure was 23.5 h/day over a period of 14 days. At the end of the exposure, interstitial (Leydig) cells were isolated from the testicles of the sham-exposed and exposed animals. The cells were cultured for 48 h in the presence or absence of 1, 10, or 100 mIU/ml human chorionic gonadotropin (hCG). The luteinizing hormone (LH) analog hCG was used to check the testosterone (T) response of the sham-exposed controls and to evaluate the possible effect of the whole-body MF exposure on the steroidogenic capacity of Leydig cells *in vitro*. Testosterone content of the culture media and blood sera was measured by radioimmunoassay (RIA). In the cultures obtained from MF-exposed animals, the hCG-stimulated T response was significanly higher (p < 0.01) compared with the sham-exposed controls, while the basal T production of cells and the level of serum T remained unaltered. No MF exposure—related histopathological alterations were found in testicles, epididymes, adrenals, prostates, and pituitary glands. The MF exposure did not affect the animal growth rate and the observed hematologic and serum chemical variables. Our results indicate a presumably direct effect of whole-body MF exposure on the hCG-stimulated steroidogenic response of mouse Leydig cells.

## INTRODUCTION

In the last decades, utilization of electricity has increased and diffused in both households and industries. The frequency of the current used in these systems is 0–300 Hz. This frequency interval is termed extremely low frequency magnetic field (ELF-MF), which is a nonionizing radiation having photon energy too weak to break the atomic bonds. One of the most important fields of research in this topic is the investigation of the possible biological effects of power-line frequency (50/60 Hz) MF. At these frequencies, male and female reproductive functions have been proposed as possibly sensitive targets for the biological actions of MF. However, experimental data on male reproduction are quite limited and contradictory.

Kato et al.[1] reported that 6 weeks of nearly continuous exposure to circularly polarized 50-Hz MF at 1, 5, or 50 μT flux densities did not change plasma testosterone (T) in rats. Margonato et al.[2] did not find any MF-induced morphologic and histologic changes in the testes of rats after prolonged exposure to a 50-Hz MF of 5 μT. In another experiment, the same working group exposed rats for 8 months (8 h/day, 5 days/week) to a 50-Hz MF of 5 or 100 μT and they also did not observed any MF-related alterations in testicular histology[3]. However, Picazo et al.[4] reported a significant increase in the size and weight of the testicles (due to an increase in the interstitial tissue) and elevated levels of serum T after a 10-week exposure of 50-Hz/100-μT MF. Sert et al.[5] also found increased T levels in rats that they left for 5 weeks, 3 h/day in a 50-Hz/0.8-mT MF. In addition, statistical difference was found between the control and exposed animals with respect to head with lack of hook and isolated head type sperm. Besides this, they observed decreased spermatogenesis in some seminiferous tubules, congestion in blood vessels of the interstitium, and increases in interstitial edema and Sertoli cells. Al-Akhras et al.[6] observed a partly reversible reduction in male rat fertility after 90-day exposure to a 50-Hz MF of 25 μT. However, this working group did not find any MF exposure–related effects on the fertility of male mice at the same parameters of exposure[7].

Heredia-Rojas et al.[8] did not observe any meiotic chromosome aberrations in spermatocytes and any changes in sperm morphology in mice following a 60-Hz, 2-mT MF exposure for 72 h or 10 days/8h daily. However, Lee et al.[9] reported an increased apoptotic rate of germ cells and a decrement in the number of well-organized seminiferous tubules in mice after a continuous 60-Hz, 0.5- or 1-mT MF exposure for 8 weeks. De Vita et al.[10] observed a statistically significant decrease in elongated spermatids obtained from rats 28 days after exposure to sinusoidal 50-Hz, 1.7-mT MF for 4 h. The authors suggested a possible cytotoxic and/or cytostatic effect of the applied MF on differentiating spermatogonia.

In our previous experiments, we found that *in vitro* exposure to sinusoidal 50-Hz, 100-μT MF was able to stimulate the basal T production of primary mouse Leydig cell culture[11].

The main goal of this study was to evaluate the possible direct effect of whole-body 50-Hz MF exposure on the *in vitro* steroidogenic capacity of cultured Leydig cells.

## MATERIALS AND METHODS

### Animals

CFLP mice (HUMAN, Gödöllő, Hungary) weighing 32–37 g (8–9 weeks old) were kept in a room with a 12:12 light/dark photoperiod, temperature of 20–23°C, and relative humidity of 50–60%. In each experiment, 10–13 sham-exposed and another 10–13 exposed mice were kept in plastic cages and allowed free access to standard laboratory pellets (Altromin) and tap water. The MF exposure was carried out in four repeated experiments.

### Magnetic Field Exposure

The sinusoidal, 50-Hz MF was generated by a pair of double-wound coils connected to an internally developed, stabilized AC-current generator. The coils were embedded in molded epoxy resin to avoid vibration. The inner diameter of the coils was 42 cm. The distance between the two parallel coils, mounted horizontally above and below the mouse cage, was 32 cm. The orientation of MF was vertical[12]. The magnetic flux density was measured with a Hall detector-type MF sensor and monitor (Lakeshore M420)

and was continuously monitored with and EMDEX II MF dosimeter. The flux density of the 50-Hz MF was 100 μT (rms) with less than ±3% inhomogeneity within the exposed animal chamber. The combined geomagnetic field was 48 μT in the laboratory. The duration of exposure was 23.5 h/day over a period of 14 days. The daily 0.5-h breaks were necessary for cleaning cages and feeding.

Body weights were recorded on the first and the last day of the treatment. Following the termination of exposure, between 10:30 and 11:00 a.m., mice were anesthetized with 60 mg/kg ip. pentobarbital (Rhone-Poulenc Rorer, Vitry sur Seine, France) and testicles, epididymes, adrenals, prostates, and pituitary glands were removed and blood samples were taken. After surgery, the animals were overdosed with pentobarbital.

## Interstitial Cell Preparation and Incubation

Interstitial cells were prepared and cultured from the right testicles of the animals. (The left testicles were used for histology.) For isolation of interstitial cells by mechanical dissociation without enzyme treatment, the method of Stoklosowa[13] was used with some modification, as described earlier[14]. In each experiment, interstitial cells were isolated and collected in two blocks: one obtained from sham-exposed and another from the exposed animals. These mixtures of interstitial cells were diluted to $10^6$ cells/ml in Eagle’s Modified Essential Medium (EMEM; Flow Laboratories, Irvine, Ayrshire, U.K.) supplemented with 10% Fetal Bovine Serum (FBS; Gibco BRL), 100 IU/ml penicillin, and 100 μg/ml streptomycin (Sigma Chemical Co., St. Louis, MO). The cell suspensions were plated (with final volume of 500 μl/well) into sterile 24-well plates (Corning Glassworks, Corning, NY). Leydig cells were identified by the histochemical reaction for 3β-hydroxisteroid dehydrogenase (3β-HSD) enzyme[15,16]. In brief, cells were incubated for 3.5 h at 34°C in the culture medium containing of 0.6 mmol/l dehydroisoandrosterone (DHA; Sigma), 0.6 mmol/l nicotinic acid adenine dinucleotide (NAD; Sigma), 0.2 mmol/l nitro blue tetrazolium (NBT; Sigma), and 7% propylene glycol. The percentage of 3β-HSD positive cells was at least 70%. To assess viability prior to adding FBS to the culture medium, samples were taken and stained with trypan blue solution (Sigma) and the cells were counted in a hemocytometer. The intersitial cells were cultured in the presence or absence of 1, 10, or 100 mIU/ml human chorionic gonadotropin (hCG; Choriogonin; Richter, Budapest, Hungary). The luteinizing hormone (LH) analog hCG was used to check the T response of the sham-exposed controls and to evaluate the possible effect of the *in vivo* MF exposure on the steroidogenic capacity of Leydig cells *in vitro*. Cell cultures were maintained for 48 h at 34°C under a humidified atmosphere of 95% air/5% $CO_2$. Following the incubation, the aliquots of the culture medium were centrifuged and the supernatant was collected and frozen at –20°C until T determination by radioimmunoassay (RIA).

## Testosterone Radioimmunoassay

Quantification of T directly from aliquots of the cell culture media was performed by RIA. In addition, blood samples were taken from randomly selected animals to check the T levels of blood sera[1,2,6,7]. [$^3$H]-testosterone (3.74 TBq/mmol) was obtained from Amersham, Buckinghamshire, U.K. The T standard and antiserum were kindly donated by Dr. Tibor Fehér (Semmelweis Medical School, 1$^{st}$ Department of Internal Medicine). The inter- and intra-assay coefficient of variations were 9.4 and 6.5%, respectively. Cross-reactivity with 5α-dihydroxytestosterone was 7.9%.

## Histological Techniques

Testes (left), epididymes, adrenals, prostates, and pituitary glands were fixed in buffered formol saline for histological examination. These tissues were dehydrated in ethanol, embedded in paraffin wax, and stained with hematoxylin and eosin.

### Hematology

Blood was taken from aorta, collected in plastic Eppendorf tubes containing $K_3EDTA$. White (WBC) and red (RBC) blood cells counts, hemoglobine concentration (HGB), volume of packed red cells (VPRC), and platelets (PLT) were determined using the Animal Blood Counter Vet abc™ (Roche).

### Serum Chemistry

At sacrifice, free-flowing blood was collected in plastic Eppendorf tubes. After centrifugation, serum was removed and the samples were then analyzed for serum alkaline phosphatase, glutamic-oxaloacetic transaminase (GOT), glutamic-pyruvic transaminase (GPT), cholesterol, and triglycerides by an auto analyzer instrument (A.L. Instruments, France).

### Statistics

The data were analyzed by Student's t-test and ANOVA using GraphPad Prism 3.01 computer software. To eliminate heterogeneity of variance among the data of experiments performed at different times, T levels of the culture media were expressed in percent of sham-exposed, *in vitro* 100 mIU/ml hCG-stimulated controls. A value of $p < 0.05$ was considered to be significant.

## RESULTS

### Testosterone Production of Interstitial Cell Culture

The Leydig cells used in all experiments were viable (>90% viability; checked by trypan blue exclusion test) and steroidogenically active. The sham-exposed mean 100-mIU/ml hCG-stimulated T response (control, 100%) was 245.21 ± 35.92 ng/$10^6$ cells. In the Leydig cells obtained from animals subjected to MF exposure, a significant elevation in hCG-stimulated T response was seen over a 48-h culture, while the basal T production and initial cell yield remained unaltered (Fig. 1). No MF exposure–related alteration was observed by phase contrast microscopy in cell size, attachment, and density of the 48-h semiconfluent monolayers.

### Serum Testosterone

Concentration of serum T was also checked in randomly selected animals (n = 19). The mean (±SEM) T level of the exposed animals (350.26 ± 47.40 pg/ml) was found not to be significantly different from the sham-exposed controls (364.89 ± 40.88 pg/ml).

### Histology

No MF exposure–related histopathological alterations were found in testicles, epididymes, adrenals, prostates, and pituitary glands.

### Body Weight

There was no significant difference in the body weights of exposed animals compared to the sham-exposed controls on the first and the last day of the exposure.

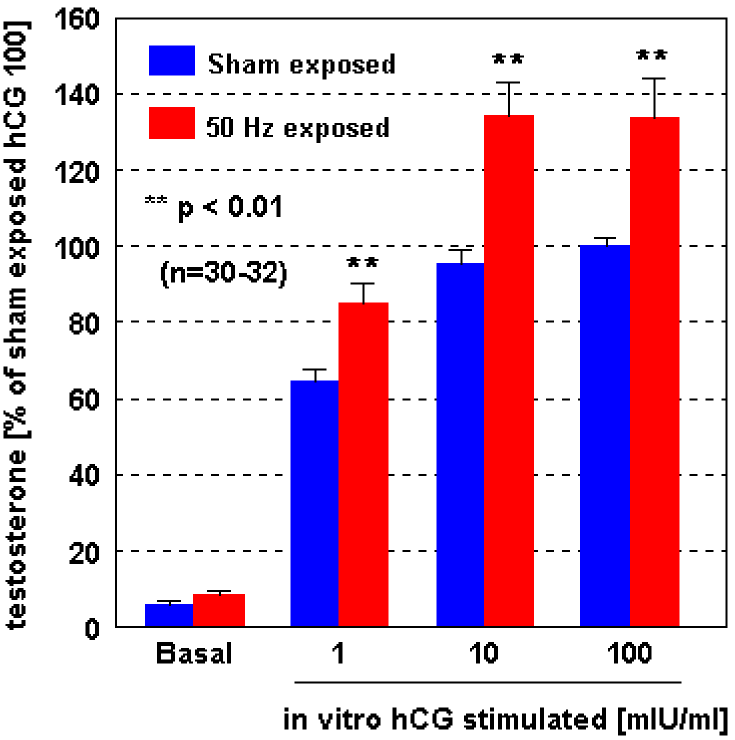

**FIGURE 1.** Effect of whole-body 50-Hz MF exposure on the basal or *in vitro* hCG-stimulated T response of 48-h mouse Leydig cell culture. Bar values represent the mean (±SEM) T production as percent of sham-exposed, *in vitro* 100-mIU/ml hCG-stimulated cells (n = 30–32) determined in four independent experiments. The statistical difference from sham-exposed, 100-mIU/ml hCG-stimulated control is indicated by ** ($p < 0.01$).

## Hematology

The hematologic variables (mean ± SEM) of exposed and sham-exposed mice are summarized in Table 1. No statististical difference was found between exposed and sham-exposed animals.

## Serum Chemistry

The mean values (± SEM) of alkaline phosphatase, GOT, GPT, cholesterol, and triglycerides for exposed and sham-exposed mice are shown in Table 2. Statistical analysis did not give evidence of any significant difference between the two groups.

**TABLE 1**
**Mean Values ± SEM of Blood Variables**

| Blood variables | Sham-Exposed | 50 Hz-exposed |
|---|---|---|
| WBC [$10^3$/µl] | 3.15 ± 0.33 (21) | 3.77 ± 0.23 (19) |
| RBC [$10^6$/µl] | 8.58 ± 0.12 (20) | 8.98 ± 0.10 (19) |
| HGB [g/dl] | 13.00 ± 0.17 (21) | 13.31 ± 0.15 (19) |
| VPRC [%] | 41.62 ± 0.55 (21) | 41.21 ± 0.50 (19) |
| PLT [$10^3$/µl] | 756.30 ± 19.92 (20) | 745.10 ± 25.01 (19) |

WBC, RBC, HGB, VPRC, and PLT in sham-exposed and 50-Hz-exposed mice; ( ) = number of animals.

**TABLE 2**
**Mean Values ± SEM of Serum Chemistry**

| Serum chemistry | Sham-Exposed | 50 Hz-exposed |
|---|---|---|
| Alk. phosph. [U/l] | 404.20 ± 22.45 (21) | 365.20 ± 20.42 (16) |
| SGOT [U/l] | 178.80 ± 10.70 (24) | 175.10 ± 11.36 (26) |
| SGPT [U/l] | 52.25 ± 3.07 (25) | 61.94 ± 4.22 (26) |
| Cholesterol [mmol/l] | 3.00 ± 0.16 (15) | 2.84 ± 0.11 (25) |
| Triglycerides [mmol/l] | 0.90 ± 0.09 (16) | 1.13 ± 0.11 (16) |

SGOT = serum glutamic-oxaloacetic transaminase; SGPT = serum glutamic-pyruvic transaminase; ( ) = number of animals.

## DISCUSSION

In toxicological experiments, *in vitro* measures of T production of Leydig cells seem to be more sensitive than *in vivo* indicators of testicular damage[17]. Determination of the LH- or hCG-stimulated T response of cells improves the sensitivity of this method.

Physiological T production in male vertebrates is stimulated by LH (or its analog hCG) through interaction with specific receptor located on the Leydig cell plasma membrane. Hormone-receptor interaction stimulates the classical second messenger pathway involved in steroidogenesis: adenylate cyclase - cAMP - protein kinase A[18]. While cAMP is a second messenger for LH in the control of steroidogenesis, other signaling systems exist that are potentially equally effective in controlling steroidogenesis. In addition, the action of cAMP requires other signaling pathways involving $Ca^{2+}$ and $Cl^-$, as well as arachidonic acid and its lipoxygenase products[19].

Our results indicate an elevated hCG-stimulated T response of Leydig cell cultures obtained from whole-body MF-exposed mice, while the basal T production of cells and the serum T level of the animals remained unaltered. These data suggest a presumably direct effect of whole-body MF exposure on Leydig cells. These results are in agreement with our previous findings about the effect of *in vitro* MF exposure on T response of mouse Leydig cells[11].

The mechanism of action of the applied MF exposure cannot be discerned from present results. The MF exposure might alter the sensitivity and/or density of LH receptors on the surface of the cell membrane of Leydig cells. It is known that the initial biochemical or metabolic state of the cell membrane, as well as the functional and supramolecular organization of its specific regions (cell contact areas, receptors), are sensitive targets of electric and electromagnetic field (EMF) exposures[20,21,22,23,24]. *In vitro* MF experiments have shown that signal transduction in receptorregulated cells is interfered by MF exposure. This was first noted at receptor level in bone cells and the proposed mechanism was that MF caused changes in receptor binding and/or activation[25]. In other experiments, MF reduced the reactivity of central dopamine D(1) receptor in rats[26], modulated serotonin (5HT1A) receptor binding to a radioactive agonist in rats[27], and increased the expression of some genes relating to cytokine receptors in HL60 cells[28].

Second messengers of signal transduction processes, mainly cAMP and $Ca^{2+}$, also seem to play a decisive role in this effect of MF exposure on steroidogenesis. A possible mechanism of action on steroidogenesis may be associated with the alterations in cAMP content and intercellular communication may be induced by the applied MF.

Schimmelpfeng et al.[29] found increased cAMP content and gap junction–mediated intercellular communication after 5 min of exposure to 50-Hz, 2-mT MF in monolayers of SV40-Swiss-3T3 mouse fibroblasts at intermediate cell density.

MF exposure can alter the local $Ca^{2+}$ concentration in different types of cells[30,31] and it is able to enhance the expression of voltage-gated $Ca^{2+}$ channels on plasma membrane of the exposed cells[32]. It is known that $Ca^{2+}$ signaling is the biological main target of MF because it has extreme sensitivity for coherent excitation by very low field energies within specific amplitude and frequency windows[33].

The results of our experiments show that whole-body MF exposure is able to increase the steroidogenic responsiveness of mouse Leydig cells to LH at a flux density that does not elicit any histopathological alterations in the investigated organs or changes in hematology and serum chemistry. The level of serum T was unaltered probably due to the feedback mechanisms of hypothalamic-pituitary-gonadal axis.

Further investigations are required to clarify the subcellular action of applied MF in Leydig cells, as well as to establish the biological significance of this phenomenon.

## ACKNOWLEDGMENTS

Thanks are due to Mrs. Mária Szívós and Mrs. Erika Sklánitz for their excellent technical assistance. The study was supported by research grants from the National Scientific Research Fund (OTKA No. T 034957) and the Committee for Health Studies Grant (ETT 160/2003) of the Ministry of Health, Social and Family Affairs in Hungary.
